# Editorial: Wildlife health consequences from environmental pollution

**DOI:** 10.3389/fvets.2024.1512871

**Published:** 2024-11-13

**Authors:** Catarina Jota Baptista, Fernanda Seixas, José M. Gonzalo-Orden, Paula A. Oliveira

**Affiliations:** ^1^Egas Moniz Center for Interdisciplinary Research (CiiEM), Egas Moniz School of Health and Science, Campus Universitário—Quinta da Granja, Almada, Portugal; ^2^Centro de Investigação das Tecnologias Agroambientais e Biológicas (CITAB/Inov4Agro), University of Trás-os-Montes and Alto Douro (UTAD), Vila Real, Portugal; ^3^Centro de Ciência Animal e Veterinária (CECAV/Al4Animals), UTAD, Vila Real, Portugal; ^4^Instituto de Biomedicina (IBIOMED), Universidad de León, León, Spain

**Keywords:** conservation, contamination, monitoring, one health, pollution, wildlife

In today's globalized and interconnected world, it is crucial to analyse global health concerns as shared between humans and nature. Various governmental and non-governmental organizations are advocating for integrated strategies that combine expertise and resources to address threats that often result in widespread consequences for population stability and health. The “One Health approach” emphasizes the intrinsic connection among humans, animals, and ecosystems (1, 2). Disciplines once primarily associated with environmental and biomedical sciences, such as toxicology, are now part of an integrated and transdisciplinary approach to preserve ecosystems and wildlife. Health risks and diseases are frequently linked to climate change and habitat destruction, leading to significant impacts on keystone species and biodiversity hotspots across the globe. It is evident that combating species extinction is unsuccessful without healthy wild habitats. Environmental hazards such as chemical pollution, and radiation severely impact the health of various species and may change multiple abiotic factors (3).

Different wild species can serve as effective sentinels and bioindicators of these health risks, alerting us to the repercussions of our environmental practices. Biomonitoring studies should be part of the wildlife research priorities due to the contributions it may give to species and ecosystems' conservation (4). In Denmark, Rasmussen et al. reported the presence of rodenticides, insecticides, and herbicides in 84, 43, and 50% of hedgehogs' liver samples (*Erinaceus europaeus*). The detection frequencies differed significantly between the Western and Eastern part of the country, which reveals that different wild populations are exposed to different levels of pesticides. Even though no correlations or significant pathologic lesions have been described, these authors suggest that peak exposure to pesticides, occurring simultaneously with chronic exposures, may lead to currently unexplored effects to hedgehogs' health.

Anthropogenic actions always have an impact in nature, even if these actions are planned to benefit wild species or to preserve natural resources. In fact, the impact of conservation and research actions should also be evaluated to provide a risk assessment of their use. From this perspective, Manville et al. discussed the benefits and health risks related to the use of radio tracking, radio telemetry, and related microchip and data-logger technologies used to study, monitor and track wildlife. In general, researchers, biologists, veterinarians sometimes use these devices without fully understanding or evaluating potential consequences to the animals. Besides presenting and discussing this matter, these authors provide a comparative analysis of this radio devices and suggested management practices to avoid potential problems. Continuing on the subject of radiation-induced pollution, the consequences of nuclear accidents and the use of nuclear energy continues to be an important matter of concern from a One Health perspective. Once again, wildlife health scientist must be part of this discussion, since radiation exposure also affects the health and stability of several wild populations. Hayama et al. analyzed the effects of radiation exposure due to the Fukushima Daiichi nuclear accident (in 2011) on the fetal growth of wild Japanese monkeys (*Macaca fuscata*). By comparing animals collected before and after the incident, these authors reported that radiation exposure due to the nuclear accident may have contributed to the delayed fetal growth, since fetal body weight and fetal head circumference growth were negatively associated with relative exposure dose rate.

Ultimately, but of no small importance, the effects of pollution in wildlife health should always be part of an integrated approach with biological and biomedical research, such as microbiology and infectious agents (5). That is one of the multiple reasons why conservation actions must include professionals from different fields, including environmental scientist, biologists, and veterinarians. The microorganisms and their interaction with environmental factors have a significant impact on animal health. In birds, intestinal fungal composition plays a crucial role in the health of the individual. Therefore, it has an impact on the conservation of endangered bird species, as suggested by Ma et al..

The diversity of Research Topics covered within this Research Topic are representative of the large scope of this scientific field. This provides an illustration of the current knowledge and potential of the subject, working as an impulse for new studies.
